# The functional analysis of Cullin 7 E3 ubiquitin ligases in cancer

**DOI:** 10.1038/s41389-020-00276-w

**Published:** 2020-10-31

**Authors:** Le Shi, Dongyue Du, Yunhua Peng, Jiankang Liu, Jiangang Long

**Affiliations:** Center for Mitochondrial Biology and Medicine, The Key Laboratory of Biomedical Information Engineering of Ministry of Education, School of Life Science and Technology and Frontier Institute of Science and Technology, Xi’an Jiaotong University, Xi’an, 710049 China

**Keywords:** Cancer, Molecular biology

## Abstract

Cullin (CUL) proteins have critical roles in development and cancer, however few studies on CUL7 have been reported due to its characteristic molecular structure. CUL7 forms a complex with the ROC1 ring finger protein, and only two F-box proteins Fbxw8 and Fbxw11 have been shown to bind to CUL7. Interestingly, CUL7 can interact with its substrates by forming a novel complex that is independent of these two F-box proteins. The biological implications of CUL-ring ligase 7 (CRL7) suggest that the CRL7 may not only perform a proteolytic function but may also play a non-proteolytic role. Among the existing studied CRL7-based E3 ligases, CUL7 exerts both tumor promotion and suppression in a context-dependent manner. Currently, the mechanism of CUL7 in cancer remains unclear, and no studies have addressed potential therapies targeting CUL7. Consistent with the roles of the various CRL7 adaptors exhibit, targeting CRL7 might be an effective strategy for cancer prevention and treatment. We systematically describe the recent major advances in understanding the role of the CUL7 E3 ligase in cancer and further summarize its potential use in clinical therapy.

## Introduction

Posttranslational modifications of proteins have been reported to affect various aspects of protein function, which in turn controls a broad range of fundamental cellular functions^1^. Cellular proteins can be degraded via the ubiquitin (Ub)–proteasome system. This precisely controlled process, mediated by three components (Ub, ubiquitination enzymes and 26S proteasome), is crucial for maintaining normal cellular functions^2^. The Ub–proteasome system pathway is associated with cancer due to its effect on the degradation of multiple tumor-related proteins^3,4^ and can uniquely regulate almost every aspect of cell biology, such as cell growth and invasion, the cell cycle, and cell metabolism^5^.

The ubiquitination of substrates is processed by three enzymes. The E3 Ub ligase is central and cooperates with an Ub-activating enzyme (E1) and a Ub-conjugating enzyme (E2); the formed complex binds to substrates via the adaptors and transfers the polyubiquitin chains from E2 onto the target proteins^6^. Numerous studies have revealed that the dysfunctions in cullin (CUL)-ring ligases (CRLs) play crucial roles in tumor growth and metastasis. CRLs are a large group of E3 ligases characterized by three important subunits: a CUL protein, a ROC1 ring finger protein, and a substrate receptor module. CUL proteins (CUL1, CUL2, CUL3, CUL4a, CUL4b, CUL5, CUL7, and CUL9) that contain a conserved CUL homology domain, are molecular scaffolds and are capable of binding to a small ring domain protein to recruit the E2 Ub-conjugating enzyme. At the N-terminus, all CUL proteins except CUL7 have three CUL repeat sequences, which interact with CUL-specific substrate-targeting molecules^7^. CUL7 is the seventh and most recently identified member of the CUL family to date. *CUL7* mutations have been reported in 3-M syndrome and Yakuts short stature syndrome, the characteristics of which are profound growth retardation and endocrine disorders, with a normal mental status^8,9^. Furthermore, CUL7 expression is increased in multiple cancers, and is associated with tumor growth, invasion, and metastasis. The diverse biological functions of CUL7 provide new molecular research perspectives.

## Introduction of the CUL7 E3 ligases

**Fig. 1:**
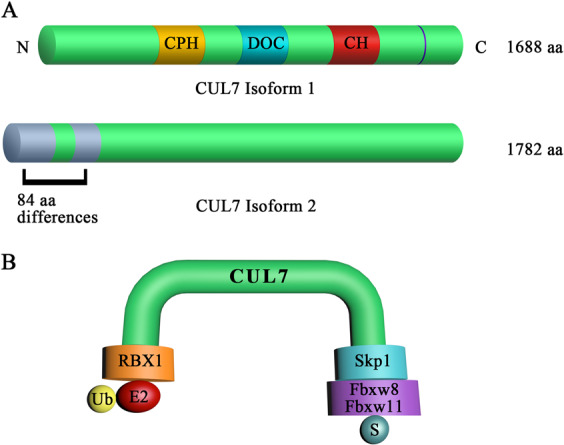
Overview of the components of CRL7. **a** CUL7 structure. The orange, blue, and red boxes indicate the main domains in CUL7. The blue vertical line is a conserved C-terminal Nedd8 conjugation site. The gray boxes indicate the aa that differs between CUL7 isoforms 1 and 2. **b** A schematic illustration of CUL7-mediated ubiquitination.

CUL7, also named as p185, was discovered by Nomura in 1994 and characteristically contains 1698 amino acids (aas) in humans^10^. Compared with canonical CUL family members, CUL7 exhibits unique characteristics. In addition to the highly conserved CUL domain used to bind ring box protein 1 (Rbx1) or Rbx2 to recruit of the E2 Ub-conjugating enzyme, CUL7 is composed of two other distinct motifs: The DOC domain is similar to that of the anaphase-promoting complex^11^, and the CPH domain is a conserved domain in CUL7, parkin-like cytoplasmic protein (PARC) and HERC2 proteins^12^. Furthermore, CUL7 has two isoforms: isoform 1, the canonical sequence, and isoform 2, which has 85 extra aas at the N-terminus (Fig. 1a). Interestingly, CUL7 assembles an S phase kinase-associated protein (Skp1)/CUL-ring/F-box (SCF)-like E3 complex that includes the component Skp1 and CUL7 and several WD40 repeat-containing F-box proteins^13–15^. Notably, CUL7 selectively interacts with the Skp1-Fbxw8 heterodimer rather than the monomeric Skp1 protein that is typically seen in SCF-type of E3 ligases^13,16,17^. Moreover, Fbxw8 (also named Fbx29, Fbw6, or Fbxw8) was historically widely believed to be the only F-box protein to bind to CUL7 via Skp1^18,19^. However, Luo et al. recently showed that CUL7 mediates activation-induced cytidine deaminase (AID) ubiquitination by forming a complex with Fbxw11 but not Fbxw8^20^ (Fig. 1b). Specifically, CUL1–Rbx1 binds to CUL7–Rbx1 via common heterodimerization with Fbxw8, enhancing the degradation of CUL1 substrates through the highly assembled complexes^13,21^.

Five cellular proteins have been previously recognized as substrates of the CUL7 E3 ligase: cyclin D1^16^, insulin receptor substrate (IRS-1)^22^, hematopoietic progenitor kinase 1 (HPK1)^23^, Golgi reassembly-stacking protein 65 (GRASP65)^24^, and TBC1 domain family member 31 (TBC1D31)^25^. Recently, additional proteins have been implicated in the degradative process of the CUL7 E3 Ub ligase: histone H2B-like (H2B)^26^, Mof4 family associated protein 1 (MRFAP1)^27^, AID^20^, caspase-8^28^, macrophage stimulating 1 (Mst1)^29^, and pleckstrin homology like domain family B member 2 (LL5β)^30^. Interestingly, several proteins, such as p53^12,31,32^, SV40 T antigen^33–35^, glomulin (GLMN)^36^, PARC^37,38^, obscurin-like 1 (OBSL1), and coiled-coil domain containing 8 (CCDC8)^39^, have been shown to interact with CUL7, but this interaction does not affect their stability, suggesting that the CUL7 E3 ligase may perform a proteolytic function and also play a non-proteolytic role.

## Physiological and pathological effects of the CUL7 E3 ligase

Embryonic lethality has been observed to be associated with CUL E3 ligases, including CUL1, CUL3, CUL4a, and CUL7^40–42^. Embryonic lethality in mice lacking CUL1, CUL3, or CUL4a occurs at the early stage of embryo formation (E4.5-E7.5) before organ formation, while mice lacking CUL7 show neonatal lethality and severe intrauterine growth retardation (IUGR) with significantly smaller foetuses and placental anomalies, indicating that the mechanisms by which CUL7 and the other three CUL E3 ligases mediate mammalian development are obviously distinct. Further studies showed that Fbxw8 is the main F-box protein that binds CUL7 via the adaptor Skp1 and that mice lacking Fbxw8 exhibited a phenotype similar to that of *CUL7*^−/−^ mice, particularly regarding growth suppression and abnormal placental development, suggesting that Fbxw8 is an important element in CUL7-regulated growth. However, *Fbxw8*^−/−^ mice do not exhibit all of the characteristics appearing in *CUL7*^−/−^ mice, the relatively mild phenotype of *Fbxw8*^−/−^ mice implies that other elements, independent of Fbxw8 function, are involved in the CUL7-based SCF-like complex^19,43^. Furthermore, depletion of *CUL7* in C2C12 cells and heterozygous knockout of either *CUL7 or Fbxw8* in mice resulted in increased IRS-1 protein levels, Akt and MEK/ERK activity, and cellular glucose uptake upon insulin stimulation^22^. These results support a model in which CUL7 regulates insulin signaling and glucose homeostasis. However, although the promitogenic signaling pathways are significantly activated, mouse embryonic fibroblasts from *CUL7*^−/−^ mice grow poorly due to the arrest of most cells in the G1 phase. Moreover, in the *CUL7*^−/−^ mouse embryonic fibroblasts, the levels of p16 and hypophosphorylated retinoblastoma protein are dramatically increased, accompanied by enhanced activation of β-galactosidase, suggesting that these cells are undergoing senescence. Of note, liver kinase B1 (LKB1), a central regulator of energy metabolism, can regulate CRL7-mediated IRS degradation, which indicates that CUL7 contributes to the regulation of energy metabolism^44^. In addition, *CUL7* heterozygous mice presented a reduced percentage of CD19^+^ B cells but a normal or a slightly elevated level of T cells. Mechanistically, *CUL7* knockout may result in high levels of AID in immature B cells and induce B cell apoptosis, leading to a low CD19^+^ B cell percentage, which provides insight into the development of immunological adjuvants for vaccines^20^. CUL7 also functions in the initiation and progression of tumors, playing an oncogenic role in the majority of tumors. For instance, *CUL7* depletion in lung cancer cells depleted of inhibits cell proliferation and xenograft tumor growth^45^, which will be further discussed in subsequent sections.

To this end, *CUL7* mutations have been reported to exist in 3-M syndrome and the Yakuts short stature syndrome, the characteristics of which are profound growth retardation and endocrine disorders, with a normal mental status^8,9^. Sequence analysis of *CUL7* in 3-M syndrome showed the presence of 25 distinct mutations in the *CUL7* gene. Among them, 19 resulted in early termination of translation, and 6 of which led to missense mutations^8,46^. Recently, several novel *CUL7* mutations have been discovered in 3-M syndrome, expanding our knowledge of phenotype-genotype correlations in this disease^47–49^. Moreover, Maksimova proposed that a founder chromosome responsible for the *CUL7* mutation also exists in Yakuts short stature syndrome^9^.

## CUL7 in cancer

Numerous studies have shown that the CUL family is associated with the initiation and progression of tumors. It has been reported that mutations in or deregulation of several members of this gene family are related to the appearance of malignant phenotypes. These alterations mainly affect substrate stability, which regulates the complicated signaling pathways in cells, controls cell proliferation and cell-cycle progression, and thus promotes tumorigenesis or suppresses tumor growth or metastasis^50,51^. CUL7 is a member of the CUL family of proteins; however, unlike that of other CUL family members, the role of CUL7 has not been studied in various types of cancer or systematically summarized.

In 2010, CUL7 was reported to be a novel gene that was potentially related to the pathogenesis and progression of hepatocellular carcinoma (HCC)^16^. Furthermore, immunohistochemical analysis revealed that CUL7 protein expression was enhanced in HCC tumor tissues compared with normal liver tissues, especially in metastatic HCC tumor tissues, and was negatively correlated with long survival. Overexpressing or knocking down *CUL7* in vitro regulates cell proliferation, growth, migration, and invasion^52–54^. Fu et al. also indicated that CUL7 was a regulator of the endothelial-mesenchymal transition (EMT) in choriocarcinoma, suggesting its underlying role in the invasion and metastasis of tumors^55^. Later, Guo et al. discovered that CUL7 exhibited higher expression in breast cancer specimens than in paired normal tissues and was involved in the invasion and migration of breast cancer cells^56^. Qiu further analyzed the clinical significance of CUL7 in breast cancer tissue, and noted the mechanisms of CUL7 in the invasion and migration of breast cancer cells^57^. Consistent with the overexpression of CUL7 in tumor tissues, a study by Xi et al. revealed that the transcriptional expression of CUL7 was also higher in epithelial ovarian cancer tissues than in normal tissues and that the CUL7 protein level was associated with lymph node metastasis^58^. A study in esophageal carcinoma (EC) showed that CUL7 protein expression was significantly higher than that in normal tissues, and was positively associated with tumor invasion and metastasis. An in vitro investigation demonstrated that CUL7 promotes the EMT of EC1 cells, indicating the mechanism of EC tumor metastasis^59^. Moreover, Zhi et al. identified that CUL7 was able to differentiate the metastatic and non-metastatic colon cancer samples by using a support vector machine classifier^60^. These studies demonstrate that CUL7 plays a crucial role in the initiation and development of several types of cancers and might be a biomarker to distinguish metastatic tumor samples.

**Fig. 2:**
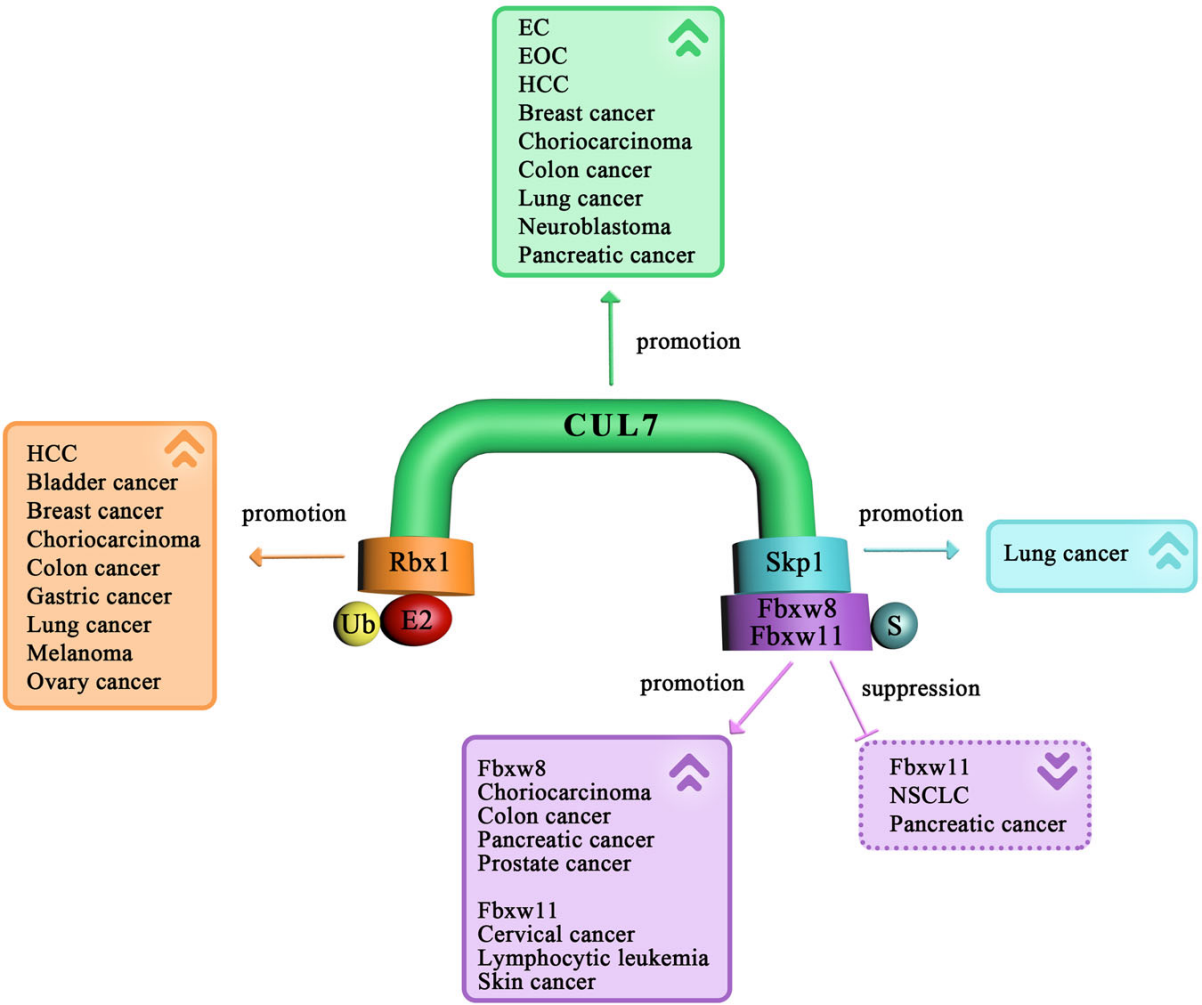
The CRL7 E3 ligase in tumorigenesis. The different colors indicate various components of CRL7. The solid box indicates that CRL7 promotes tumor growth, invasion, or metastasis in several types of cancers. The faint box indicates a potential tumor-suppressive role for CRL7. EOC, epithelial ovarian cancer.

Additionally, Kim et al. revealed that CUL7 regulated the cell growth in human neuroblastoma and that the transcriptional expression of CUL7 was upregulated in non-small cell lung cancer (NSCLC)^61^. Men et al. further discovered that CUL7 protein expression was increased in human primary lung cancer tissues and that knocking down CUL7 expression in lung cancer cells inhibited cell proliferation and xenograft tumor growth^45^. Moreover, CUL7, as the scaffold protein in the E3 ligase complex, promotes the proliferation of pancreatic cancer cells, suggesting a potential oncogenic role in pancreatic cancer^23^. To date, the effect of CUL7 on the invasion and metastasis of neuroblastoma, lung cancer, and pancreatic cancer is unknown, suggesting that further research is still needed to explore the relationship between CUL7 and the progression of these tumors. To our knowledge, CUL7 has been studied in several types of tumors, and the studies suggest that CUL7 promotes the cell growth, invasion, and metastasis in most of these tumors, might play an oncogenic role in cancer and has the potential to be used as a biomarker for the prognosis of metastatic cancers (Fig. 2).

These studies showed that CUL7 promotes cell growth and migration. In contrast, studies have revealed that CUL7 may have the opposite effect and suppress cell growth. DeCaprio et al. discovered that CUL7 had a potential tumor-suppressive role in viral transformation by using an SV40 T antigen model^35^. Furthermore, the mTOR/IRS-1 negative feedback loop was related to the inhibition of malignancy by restraining PI3K activity^62,63^. IRS-1, the critical molecule downstream of the insulin and the insulin-like growth factor 1 receptor^64^, can be ubiquitinated by the CUL7 E3 ligase, suggesting that such tumor-suppressive activity might be related to CUL7-mediated targeted degradation of IRS-1. Recently, Sun et al. revealed that CD36 could negatively regulate insulin activation, indicating that CD36 interacted with IRS-1, thereby abrogating the binding between IRS-1 and CUL7, which would further increase IRS-1 stability and affect insulin signaling^65^ and might ultimately result in tumor suppression. In addition, the level of the voltage-gated potassium channel Eag2, which promotes cell migration in medulloblastoma^66,67^, is dramatically decreased by CUL7 overexpression^68^, which might also suppress the progression of tumors. Therefore, CUL7 may play a suppressive role in tumor growth and metastasis. However, the dual, context-dependent effects of CUL7 in growth promotion and retardation remain to be further explored.

## The ring box protein Rbx1, adaptor Skp1, and F-box proteins Fbxw8 and Fbxw11 in cancer

As previously described, CUL7 might be an oncoprotein in the majority of cancers and could form a complex with Rbx1, Skp1, and the F-box. Similar to the CUL7 scaffold proteins, the catalytic components Rbx1 and Skp1 are also regarded as an oncoprotein in most of the cancers. Thus, dysregulation of these genes will regulate the oncogenic phenotype of CUL7. Of note, Rbx1 could also interact with CUL1, CUL2, CUL3, and CUL4, and Skp1 could also bind to CUL1. Thus, the alterations in Rbx1 and Skp1 that regulate the phenotype of CUL7 are not specific. However, given that the consistent regulation of the oncogenic phenotype, dysregulation of these components may strengthen CUL7-targeted treatment.

Rbx1 is an important component of the E3 Ub ligase SCF protein complex. Rbx1 interacts with the scaffold CUL protein and transfers Ub to substrates^69,70^. Overexpression of Rbx1 has been detected in various tumor tissues, including in breast, ovarian, bladder, liver, gastric, lung, and colon cancer, and in numerous cancer cell lines^71–74^. It has also been shown that Rbx1 is related to invasion and metastasis in gastric and lung cancer tumor tissues, and the downregulation of Rbx1 expression suppresses the cell proliferation, migration, and metastasis in the two cancer cell lines and bladder and melanoma tumor cells^75–80^, which may be a therapeutic strategy in cancer treatment and a potential marker for evaluating the treatment outcome (Fig. 2). However, there have been limited studies in genetically engineered animal models, and the mechanistic function of Rbx1 in different cancers remains to be elucidated.

Numerous lines of evidence demonstrate that SCF complex components promote the ubiquitination and degradation of regulatory proteins and play roles in tumorigenesis and progression^4,81^. The function of Skp1 is very important in cellular activities, including the ubiquitination of cell-cycle-related proteins^82^, kinetochore function, cell division^83,84^, and tumor formation^85^. Liu et al. revealed that the expression of Skp1 was upregulated in 36/64 (56.3%) of NSCLC samples, while patient prognosis was negatively related to the Skp1 protein levels^85^ (Fig. 2). Moreover, accumulating evidence indicates that Skp1 is associated with the formation of SCF complexes in the occurrence and metastasis of some cancers^51,86,87^. However, the expression of Skp1 has been detected in a limited number of cancers, and the mechanisms have not been fully elucidated; therefore, further exploration of the role of Skp1 in tumorigenesis and metastasis is needed.

To date, sixty-nine F-box proteins have been identified in humans^88^. The F-box protein Fbxw8 is also known as Fbw6 or Fbx29 and interacts with CUL7 at its N-terminal F-box domain. The C-terminus of Fbxw8, which specifically binds to substrates, contains a WD40 repeat domain^36^. Interestingly, CUL7 interacts with only two F-box proteins, including Fbxw8, and binding is linked by the adaptor Skp1^8,18^. Studies have shown that Fbxw8 is involved in cancer cell growth via proteolysis^89^. Consistent with the finding that CUL7 participates in the promotion of migration and invasion in choriocarcinoma cells^55^, Fbxw8 expression levels are increased in human trophoblast cell lines, including choriocarcinoma^90^, and regulate cell proliferation^23,89–91^ (Fig. 2), while the effect of Fbxw8 on the tumor migration, invasion, and metastasis remains unclear, indicating that more research about the impact of Fbxw8 on cancer progression is needed.

β-Transducin repeat-containing protein (β-TrCP) is a member of the Fbxw subfamily of the F-box proteins. Human cells contain two different isoforms of the β-TrCP protein: β-TrCP1, also named BTRC, and β-TrCP2, also named Fbxw11^92^. It is known that Fbxw11 typically cooperates with CUL1 to form an SCF ubiquitin E3 ligase and plays a fundamental role in the regulation of cell-cycle progression and tumorigenesis. A recent study showed that Fbxw11 is another F-box protein that interacts with CUL7 E3 components, mediates the degradation of AID, and further regulates IgA class switching in a mouse B lymphocytes^20^. To date, no study has reported the effect of the interaction of CUL7 and Fbxw11 in cancer. Wang et al. revealed that the Fbxw11 protein level was aberrantly upregulated in patients with lymphocytic leukemia, and the effects were mediated by the stimulation of cell-cycle progression and activation of the NF-κB and β-catenin/TCF signaling pathways rather than the induction of apoptosis^93^. Moreover, the Fbxw11 protein level is increased in mouse skin tumors and promotes cell growth^94^, migration, and invasion^95^ through activation of the NF-κB signaling pathway. In contrast, Fbxw11 protein levels are markedly decreased in NSCLC tissues compared with those in adjacent normal tissues. Interestingly, decreasing the level of Fbxw11 dramatically promotes cell proliferation and invasion in NSCLC cells^96,97^ and pancreatic cancer cells^98^ (Fig. 2). These studies show that Fbxw11 regulates tumor cell proliferation and invasion, but the effect of Fbxw11 in different tumor types is not the same, indicating that Fbxw11 might be an oncogene or a tumor suppressor and play a critical role in tumor growth and metastasis. However, the role of CUL7 in these types of cancers is not clear, and whether CUL7 interacts with Fbxw11 in these cancers needs to be further explored. If these two proteins interact, we hypothesize that the Fbxw11-mediated regulation of the cell cycle and the NF-κB signaling pathway might be related to the interaction of Fbxw11 with CUL7 in these cancers.

## CUL7 substrate proteins in development and cancer

### Proteolytic substrates

**Fig. 3:**
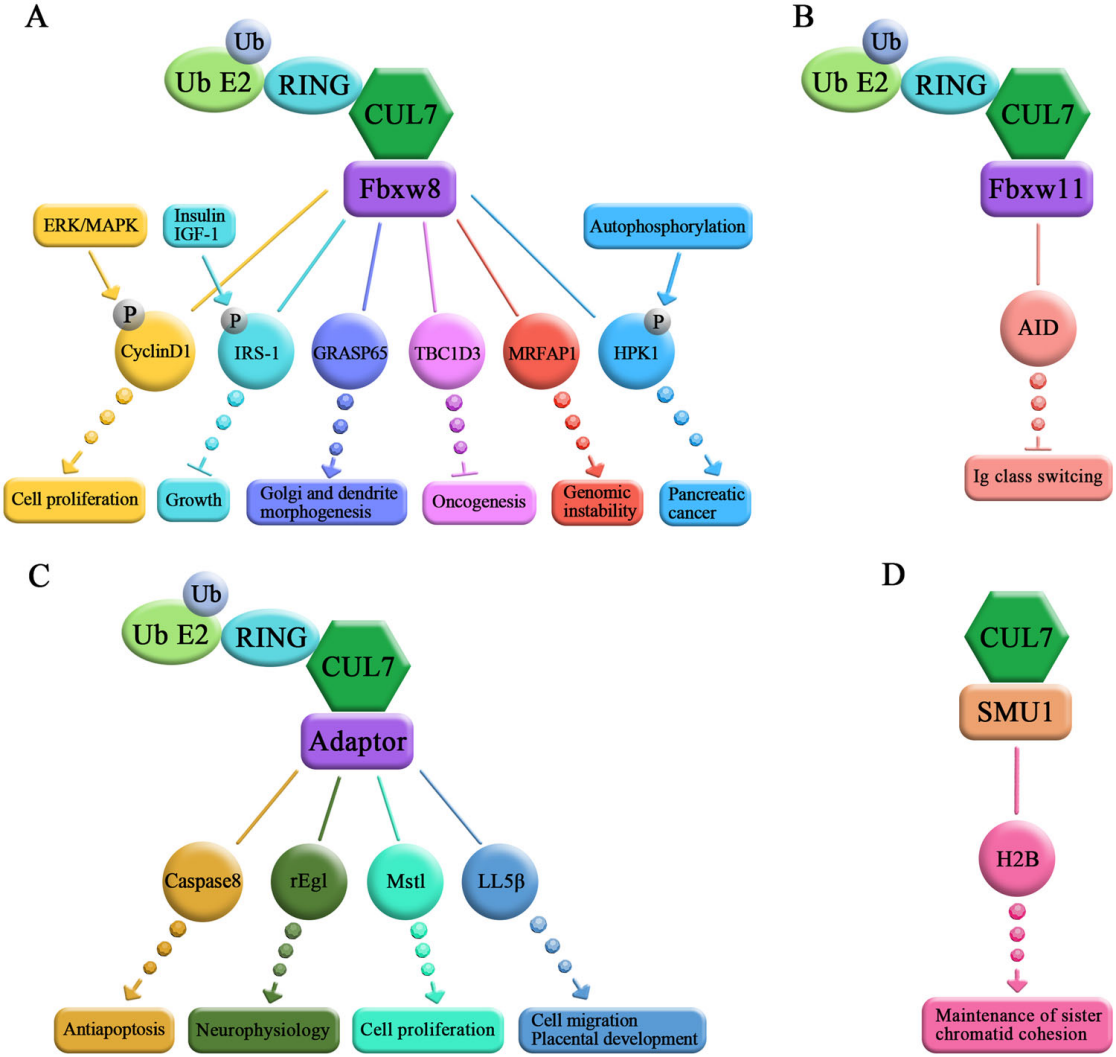
Proteolytic substrates of the CUL7 E3 ligase. **a** Proteins that have been identified as downstream ubiquitination substrates of CUL7/Fbxw8. **b** The downstream ubiquitination substrates of CUL7/Fbxw11. **c** The F-box protein-independent downstream ubiquitination substrates of CUL7. **d** The downstream ubiquitination substrates of CUL7/SMU1.

CUL7 is a member of the CUL subfamily of E3 ligases family that regulates the ubiquitination of substrates and further degrades the tagged proteins via the 26S proteasome^16^. Compared with other CUL family members, the reported substrates of CUL7 are very few. To date, Fbxw8 and Fbxw11 are the only two F-box proteins known to participate in the E3 ligase complex composed of scaffold protein CUL7, the adaptor Skp1, and Rbx1^8,18,20^, and ~6 substrates have been identified as ubiquitinated proteins that are downstream of CUL7/Fbxw8 (Fig. 3a).

Cyclin D1 is a pivotal regulator of the G1–S-phase cell-cycle transition and is overexpressed in various malignant neoplasms^99^. Okabe et al. revealed that CUL7/Fbxw8 promotes the ubiquitination of cyclin D1 in a cyclin D1^T286^ phosphorylation-dependent manner and that the degradation is related to cancer cell proliferation^89^. However, mice *Fbxw8* depletion does not exhibit the ubiquitination and subsequent degradation of cyclin D1, indicating that other E3 ligases potentially participate in the degradation of cyclin D1 via the 26S proteasome^100^. Interestingly, Lin et al. demonstrated that the SCF^FBX4-aB-cystallin^ complex could ubiquitinate cyclin D1 and that the degradation occurred in a T286 phosphorylation-dependent manner by glycogen synthase kinase 3^101^, which further supported this hypothesis, in a mouse model. IRS-1 is a major downstream effector in the insulin and insulin-like growth factor 1 signaling pathway^64^. Xu et al. found that the CUL7/Fbxw8 controlled the ubiquitination and further degradation of IRS-1 in an mTOR-dependent, negative manner and that downregulation of CUL7 and Fbxw8 resulted in the accumulation of IRS-1. In addition, Wang et al. revealed that the CUL7/Fbxw8 E3 ligase regulated the ubiquitination and targeted degradation of HPK1, which inhibited HPK1 activation and promoted pancreatic cancer cell proliferation, and the ubiquitination of HPK1 was mediated by its kinase activity in a phosphorylation-dependent manner^23,102,103^.

Litterman et al. found that CUL7/Fbxw8 was localized to the Golgi apparatus in neurons and that this localization was regulated by the cytoskeletal adaptor protein OBSL1. Furthermore, they found that CUL7/Fbxw8 interacted with, ubiquitinates and further degraded GRASP65, which regulates the morphology of the Golgi, leading to the alterations in dendritic arbours in neurons^24^. TBC1D31 plays an oncogenic role in the progression of cancer and is a hominoid-specific gene. The expression of TBC1D31 accelerates the proliferation of cell growth by activating signal transduction^104,105^. In addition, the expression of TBC1D31 is upregulated in prostate, breast^106^, and bladder cancer^107^. Kong et al. revealed that TBC1D31 was depalmitoylated, linked to lipid rafts, and degraded after CUL7-mediated ubiquitination, thus inhibiting growth factor receptor expression^108^. MRFAP1 is expressed only in mammals and is highly conserved among diverse species. MRFAP1 regulates normal histone modification levels, the activity of transcription factors involved in the Mas-related gene family and spermatogenesis^109,110^. Further study revealed that the CUL7/Fbxw8 E3 ligase ubiquitinated and further degraded MRFAP1 during the mitotic anaphase-telophase transition and that the protein level was increased in mitotic metaphase, which prevented genomic instability^27^ (Fig. 3a).

Interestingly, CUL7 may interact with and ubiquitinate proteins independent of Fbxw8^43^. A recent study revealed that Fbxw11 was a novel F-box protein involved in CUL7-mediated ubiquitination. Luo et al. revealed that CUL7 formed a complex with Fbxw11, ubiquitinated AID, and regulated the Ig class switch recombination in B lymphocytes^20^ (Fig. 3b). Kong et al. demonstrated that CUL7 promoted the ubiquitination of caspase-8 and inhibited the activation of the death-inducing signaling complex, thereby increasing the survival of cancer cells in a manner mediated by tumor necrosis factor-related apoptosis-induced ligands^28^. This group also revealed that Fbxw8 did not participate in the caspase-8 ubiquitination by CUL7^28^ (Fig. 3b). Moreover, Eag1 potassium K^+^ channels are highly expressed in the mammalian brain. Human Eag K^+^ channel mutations exist in diseases characterized by congenital neurodevelopmental anomalies^111–114^. Hsu et al. found that CUL7 was a novel binding partner of rat Eag1 and targeted the rEag1 potassium channel for proteasomal- and lysosomal degradation^68^. However, whether F-box proteins mediate the degradation of rEag1 has not been reported. In addition, CUL7 participates in Mst1 ubiquitination to induce YAP signaling and cardiomyocyte proliferation^29^. Wang et al. identified a signaling pathway controlling cell migration and placental development involved in the progression of 3-M dwarfism. This group showed that phosphorylated CCDC8 interacted with OBSL1 and that CUL7 formed a Ub ligase complex with the former two proteins in 3-M and regulated the degradation of LL5β like domain family B member 2^30^ (Fig. 3c). In addition, Shah et al. demonstrated that CUL7 could form a novel E3 ligase complex with SMU1, DDB1, and RNF40, recognizing H2B via SMU1, and further degrading H2B via the monoubiquitylation at the K120 residue, a process that maintains the cohesion of sister chromatids during mitosis^26^ (Fig. 3d). Interestingly, DDB1 is typically an adaptor protein for CRL4, and plays a similar role to that of Skp1 in the context of CRL1 complexes. In fact, proteins, such as DCAF, COP1, and CRBN, were previously shown to be the recognized substrates of CRL4^115^. Therefore, in future studies, it would be interesting to examine whether DCAF participates in the assembly of the CRL7 E3 ligase to regulate the functions attributed to CUL7. Normally, Skp1-Fbxw8 interacts with the N-terminus of CUL7, and the complex subsequently mediates the degradation of substrates^38^. Interestingly, these studies reveal that CUL7 can form novel E3 Ub complexes independent of Fbxw8.

### Non-proteolytic substrates

**Fig. 4:**
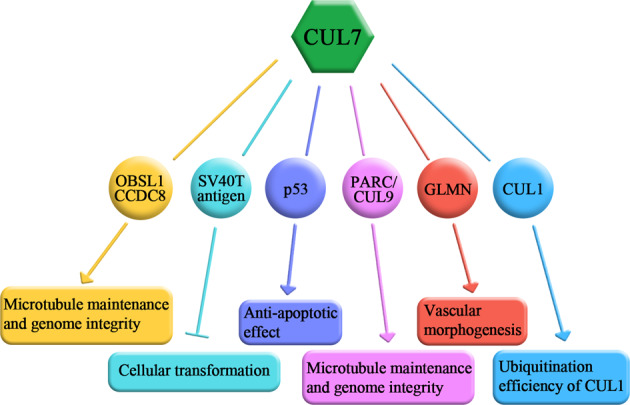
Non-proteolytic substrates of the CUL7 E3 ligase. Proteins that have been identified as binding substrates of CUL7, but are not degraded by CUL7.

Many studies have demonstrated that CUL7 interacts with several substrates, but does not degrade these binding substrates via ubiquitination, suggesting that although CUL7 has proteolytic functions, it may also play a non-proteolytic role (Fig. 4).

The CUL7, OBSL1, and CCDC8 genes are exclusively mutated in 3-M syndrome. CUL7 was found to form a complex with OBSL1 and CCDC8 but did not affect the degradation of these two complex components. This complex controls microtubule maintenance, genomic integrity, and the progression of normal growth^39^. SV40, a member of the Polymaviridae family of DNA viruses, can transform and immortalize mammalian cells in culture and induce tumors in rodents. DeCaprio et al. revealed that the binding of CUL7 and the T antigen was associated with SV40-mediated transformation. While the mutation at the residues 69–83 of the T antigen dramatically decreased the transformation capacity, the transformation potential was rescued by *CUL7* depletion, suggesting that at least in the presence of the T antigen, CUL7 may function as a tumor suppressor^35^. Interestingly, the T antigen may, as a partner of CUL7, compete with the binding between the CUL7 E3 ligase and its substrates, further regulating the degradation of targets. Zhao et al. showed that the T antigen affected the proteolytic effect of CUL7 on the Mre11-Rad50-Nbs1 complex via mutual interactions and consequently mediated the DNA damage response pathway^116^.

A previous study showed that overexpression of CUL7 promoted apoptosis in NIH-3T3 cells due to the integrity of the CUL7 BH3 domain in the presence of the SV40 T antigen^34^. However, an increasing number of studies have shown that CUL7 exerts an antiapoptotic effect in both p53-dependent and p53-independent manners. The results of nuclear magnetic resonance studies showed that CUL7 directly interacted with p53 at its CPH domain, affecting both the transcriptional activity and subcellular localization of p53^12^. Further analysis revealed that the levels of CUL7 were increased after DNA damage and inhibited the apoptosis via the p53-dependent pathway^31^. Notably, p53 was not found to be ubiquitinated or degraded in the presence of the combined expression of CUL7/Fbxw8 in vivo^31,32^. However, downregulation of CUL7 increased the protein level of p53 in neuroblastoma SHEP cells, breast cancer BT474 cells, and lung cancer cells and inhibited cell growth and invasion in cancer cells^56,61,117^. To date, no studies have reported that CUL7, as an E3 ligase, participates in the ubiquitination and subsequent degradation of p53, while MDM2 enhances the completion of p53 polyubiquitination^31,32^. These results indicate that the proteins upstream of p53 might be the substrates of CUL7. Interestingly, CUL7 plays an antiapoptotic role independent of p53. The mutation in aa 1152 of CUL7, which was considered to impede in the major activity of CUL7, inhibited apoptosis induced by MG132 and etoposide in U2OS cells, and this regulation was not associated with the binding of CUL7 and p53^117^. Thus, the clarification of the relative roles of CUL7 and p53 and the exploration of the mechanism of CUL7 in apoptosis need further attention.

PARC is associated with p53, a member of the mammalian CUL family, and is highly homologous to CUL7 with over 60% sequence identity in several domains^118^. Sequence analysis suggests that PARC may be an evolutionary precursor of CUL7^37^. CUL7 and PARC both contain CPH, DOC (DOC1/APC10), and CUL homology domains^118^, and share regions of homology with HERC2, which are associated with E3 ligase functions^18^, suggesting that PARC may be another E3 ubiquitin ligase^12^. However, PARC fails to bind Skp1 or F-box proteins, including Fbxw8^38^. Due to the sequence similarity, the N-terminus of PARC interacts with the C-terminus of p53^119,120^. However, although PARC may serve as an E3 ligase, it has no significant effect on p53 stability^37^. PARC acts as a cytoplasmic anchor protein in p53-related protein complexes to directly interact with the subcellular localization of p53 directly, and to prevent p53-dependent cell growth arrest and cell apoptosis^119^. In this context, CUL7 does not sequester p53 in the cytoplasm; unlike PARC, CUL7 promotes cell growth by antagonizing p53 functions^32^. Skaar et al. showed that CUL7 and PARC could form homodimers with themselves (CUL7-CUL7, PARC-PARC), as well as heterodimers (CUL7-PARC), and dimers containing PARC were capable of binding to p53^38^. Unlike CUL7, the PARC dimers were further found to not be unassociated with mouse viability, suggesting that CUL7 and PARC exert their own distinct functions^37^. It was also found that the CUL7-PARC heterodimer could bind p53 and form a complex^117^, and this heterodimer exhibited E3 ubiquitin ligase activity in vitro, but p53 was not required for the dimerization of CUL7 and PARC^38^. In addition, the presence of PARC and CUL7 in large (>1 mD) complexes^119^ increased the number of potential targets for CUL7-mediated ubiquitination through protein–protein interactions^117^. Moreover, Li et al. revealed that CUL7 and CUL9 (formerly PARC) could form a heterodimer and play an important role in maintaining microtubules and genomic integrity mediated by survivin^121^.

In addition, GLMN has been identified as a gene related to familial glomuvenous malformation, which is characterized by impaired vascular formation^122^. Interestingly, GLMN has been proven to interact with the Skp1-CUL7-Fbxw8 complex, indicating that the CUL7 complex plays a crucial role in vascular morphogenesis^36^. However, whether CUL7 degrades GLMN remains unknown. Notably, CUL7 interacts with CUL1 via the linker Fbxw8^13,43^. This complex does not affect CUL1 stability but increases the ubiquitination and subsequent degradation of the SCF substrate due to the relatively increased protein level of Rbx1 proteins.

### Non-interacting proteins

These above studies show that CUL7 is associated with both proteolytic and non-proteolytic functional interactions. Notably, a recent study revealed that CUL7 could regulate the ERK-SNAI2 signaling pathway and promote EMT in EC^59^. However, whether CUL7 interacts with SNAI2 has not been studied.

These studies of CUL7 substrate proteins show the potential mechanisms of CUL7 in development and tumor growth. However, only a few studies have reported which substrates are involved in the effect of CUL7 on tumor metastasis. Thus, further studies should explore the novel substrates of CUL7, especially substrates associated with cancer.

### CRL7 as a therapeutic target in cancer and metastasis

**Fig. 5:**
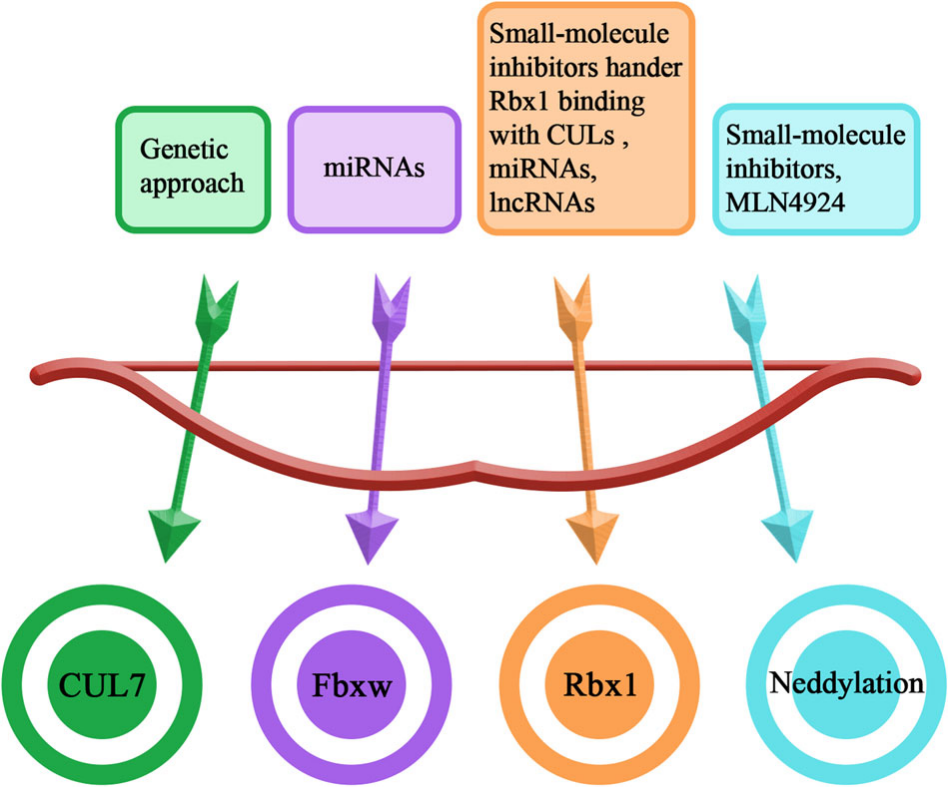
CRL7 E3 ligase inhibitors. The different colors indicate various components of CRL7. The solid box indicates potential therapy targeting CRL7 components.

CUL family proteins are closely linked with DNA replication, development, and cell-cycle control^123^. CUL proteins are abnormally expressed in various human tumor tissues and associate with extremely poor patient prognosis^124,125^. As discussed above, CUL7 is one key factor in several cancers growth and metastasis, blocking the CUL7 ligase might be an effective therapeutic strategy for malignant cancers and their progress. Most CUL proteins do not contain a classical active site, and currently, no studies have addressed inhibitor targeting CUL7. Studies revealed that knocking down *CUL7* inhibited cell proliferation, growth, invasion, and metastasis in cancer cells. Thus, genetic approach is worth considering, while mouse double knockouts of *CUL7* show neonatal lethality, indicating a partial reduction of CUL7 might be more beneficial (Fig. 5).

As scaffolding proteins, CUL family proteins bind to the E2 via their C-terminus and interact with substrates via their N-terminus, mediating the transfer of ubiquitin from E2 to the substrates. Thus, the therapeutic SCF E3 ligase inhibitors should be targeted at preventing the interaction between E3 and the substrate proteins. CRL7 comprises of CUL7, Skp1-Fbxw8, and Rbx1, and dysfunction of any component will affect the binding between E3 and the substrate. Given that CRL7 is involved in the progression of cancer cell growth, invasion and metastasis, blocking signaling maybe crucial in drug discovery for the development of therapeutic strategies. Therefore, targeting the CRL components of CUL7 is potentially relevant for drug discovery.

Fbxw8 or Fbxw11 can interact with Skp1, Rbx1, and CUL7 to form an E3 ligase complex and control the specific binding of CRLs and their substrates. In addition, a large number of F-box proteins have been identified as being involved in cell proliferation, cell differentiation, and cell-cycle regulation and are associated with tumorigenesis^8,18^. Therefore, dysregulation of Fbxw8 or Fbxw11 might be an effective therapeutic approach for CUL7-related cancers. miRNAs are noncoding single-stranded RNAs that regulate the expression of multiple genes by suppressing the translation or reducing the stability of mRNAs. Therefore, miRNA activity may participate in a variety of cellular processes, such as proliferation, differentiation, and apoptosis. Numerous studies have demonstrated that miRNAs also contribute to the initiation and development of various human cancers^126,127^. miR-218 may be an effective therapeutic target for human choriocarcinoma cell growth^128^ and miR-3160-5p suppressed the proliferation of DU145 prostate cancer cells and was negatively related to the expression of Fbxw8^91^. For Fbxw11, miR-106b-25, and miR-182 regulated the cell proliferation, migration, and invasion in NSCLC by targeting Fbxw11^96,97^. In addition, miR-182 via Fbxw11 also affected the cell growth and metastasis of pancreatic cancer^98^. The results of these studies suggested that miRNA might play a vital role in the suppression of solid cancers (Fig. 5). However, RNA-based therapeutic inhibitors frequently have off-target effects, and the delivery systems need further exploration. Notably, Zhang et al. showed that miR-182/Fbxw11 was involved in the effect of long noncoding RNA (lncRNA) PCGEM1 on cervical cancer, which suggested that lncRNA targeting Fbxw11 was a novel therapeutic strategy for CUL7-related cancers^95^.

Rbx1 is an indispensable, important component of the CRL7 E3 ligase complex^129^, and its protein levels are upregulated in a variety of cancers, such as breast, liver, kidney, and lung carcinomas^130^. Moreover, Rbx1 is required for the ligase activity of CRLs^131^. Thus, specific inhibitors preventing the binding of CUL7 with Rbx1 may be a novel therapeutic option for suppressing CUL7-related cancers. Shafique et al. demonstrated that CUL family proteins contained a conserved motif (VLYRLWLN) for binding of Rbx1. They performed detailed binding analysis with C64 and proved that it was a more potent inhibitor than the other identified inhibitors targeting the Rbx1 binding site in CUL family proteins and further hindering the degradation of tumor-suppressive substrates, suggesting that the Rbx1 peptide was a possible inhibitor of the E3 ligase complex^132^. Furthermore, several studies revealed that miRNA or lncRNA targeting Rbx1 could regulate tumor growth, invasion, migration, and metastasis, including miR-194, miR-378, miR-1827, and lncRNA LUCAT1^76,78,80^. These results provide evidence validating that targeted inactivation of Rbx1 may be an efficient therapeutic strategy for various tumors with CRL activation (Fig. 5).

The C-terminus of each CUL protein contains a conserved lysine residue, which is readily neddylated. Neddylation is the covalent addition of NEDD8 to the conserved lysine site in CUL family proteins and is mediated by three ligases. CRL activity has been proven to be closely related to and to require the CUL neddylation^133^. MLN4924 is an effective inhibitor of the E1 NEDD8-activating enzyme, selectively prevents the degradation of several distinctive substrates modified by CRLs, inducing less toxicity than targeting proteasomes by bortezomib, which universally inhibits protein degradation^134^. In fact, MLN4924 has been identified as a feasible inhibitor for the treatment of various cancers and has completed phase I clinical trials^135,136^ (Fig. 5). However, a few mutations were found in the E1 NEDD8-activating enzyme that make human tumors resistant to MLN4924^137,138^. Thus, future efforts to identify specific inhibitors are needed.

## Conclusion

In summary, CRLs are the largest family of E3 Ub ligases, forming CUL-based protein complexes for ubiquitinating and subsequently degrading the multiple substrates^139,140^. CUL7 is the CRL member that may have dual effects, either degrading or not degrading its substrates. CUL7 exhibits increased expression in different human cancers, participates in tumor growth, invasion, and metastasis, and is associated with clinical stage and prognosis. However, the role of CUL7 is explored in limited cancers and the mechanism of CUL7 is poorly elucidated, and studies on therapeutic targeting of CUL7 are lacking. We have only discussed the potential genetic therapy targeting CUL7, and the use of miRNAs targeting Fbxw8 or Fbxw11, inhibitors targeting Rbx1, and neddylation for repressing the degradation of tumor suppressor proteins. While CUL7 also exerts a tumor-suppressive effect, as partially proven by the degradation of IRS-1, which is negatively mediated via the mTOR pathway. In addition, CUL7 performs non-proteolytic functions, it regulates its substrates without binding Fbxw8 or Rbx1. Therefore, future studies may be focused on the function of CUL7 in various types of cancer growth and metastasis and the detailed mechanism that controlling the interactions between the CUL7 and its complexes, thereby improving the progression of pharmaceutical drugs development via the useful information gleaned.
